# Sex/gender differences in lifetime dementia risk among Asian American and White older adults

**DOI:** 10.1038/s44400-025-00038-8

**Published:** 2025-10-13

**Authors:** L. Paloma Rojas-Saunero, Yingyan Wu, Yixuan Zhou, Eleanor Hayes-Larson, Gilbert C. Gee, Ron Brookmeyer, Holly Elser, Alexander Ivan B. Posis, Alka M. Kanaya, Rachel A. Whitmer, Paola Gilsanz, Elizabeth Rose Mayeda

**Affiliations:** 1 Department of Epidemiology, University of California, Los Angeles Fielding School of Public Health, Los Angeles, CA 90095 USA; 2 Department of Biostatistics, University of California, Los Angeles Fielding School of Public Health, Los Angeles, CA 90095 USA; 3 Leonard Davis School of Gerontology, University of Southern California, Los Angeles, CA 90089 USA; 4 Department of Community Health Sciences, University of California, Los Angeles Fielding School of Public Health, Los Angeles, CA 90095 USA; 5 Department of Neurology, University of Pennsylvania, Philadelphia, PA 19104 USA; 6 Department of Public Health Sciences, University of California, Davis School of Medicine, Davis, CA 95616-5270 USA; 7 Division of General Internal Medicine, University of California, San Francisco, San Francisco, CA 94143 USA; 8 Department of Neurology, University of California, Davis School of Medicine, Sacramento, CA 95817 USA; 9 Division of Research, Kaiser Permanente Northern California, Pleasanton, CA 94588 USA

**Keywords:** Diseases, Health care, Medical research, Neurology, Risk factors

## Abstract

Evidence on differences in dementia risk by sex and gender is mixed. We aimed to compare lifetime dementia risk by sex/gender among Asian American and non-Latino White adults aged 60 and older. We included Chinese (*n* = 6415), Filipino (*n* = 5020), Japanese (*n* = 3314), South Asian (*n* = 1061), and non-Latino White (*n* = 143,667) Kaiser Permanente Northern California members aged ≥60 years who completed health surveys (2002–2020) and were dementia-free at baseline. We estimated cause-specific cumulative dementia incidence from age 60 to 95 years (i.e., lifetime dementia risk, treating death as a competing event) and evaluated sex/gender differences. Lifetime dementia risk was higher among women in all groups, ranging from 7 (95% CI: 2–13) percentage points higher for Japanese women vs. men to 21 (8–38) percentage points higher for South Asian women vs. men. Variations of sex/gender differences across racial and ethnic groups are potentially driven by dementia-free mortality and social and structural factors.

## Introduction

The Asian American population is the fastest-growing racial and ethnic group in the U.S., and California has the largest population of Asian American older adults in the U.S.^1^. Asian Americans are a diverse group with wide-ranging historical, cultural, immigration, and socioeconomic experiences that shape their health^2^, yet they are often aggregated in health research^3^. The “model minority myth,” a stereotype that inaccurately portrays Asian Americans as universally advantaged in health and socioeconomic status^4^, has obscured significant health disparities between ethnic groups^5^. While studies suggest that Asian American older adults have lower dementia incidence compared to other racial and ethnic groups^6^, incidence varies across Asian American ethnic groups^7–9^. Asian American women also face additional interpersonal and societal discrimination linked to adverse health outcomes^10–12^, which may increase their dementia risk.

More women than men are living with dementia^13^. Women’s higher life expectancy^14^, sex-linked biological factors^15,16^, and structural factors (e.g., educational, occupational, and financial inequities, discrimination, and barriers to healthcare)^15–17^ are thought to contribute to this difference. However, evidence on the extent to which dementia risk differs by sex and gender is mixed: some studies show higher dementia risk and poorer cognitive outcomes among women versus men, particularly at older ages (e.g., age 85 and older)^18–22^, but others do not report such differences^23–25^. A growing body of evidence suggests that sex and gender differences in dementia risk may vary across cultural and societal contexts^25–27^. However, most U.S. studies have predominantly focused on the non-Latino White population, and to a lesser extent, Black and Latinx populations^17–19,22,28^. Two studies have reported higher dementia incidence rates in Japanese American and Filipino American women versus men^29,30^, although incidence rates (cases/person-time) do not explicitly account for dementia-free mortality as a competing event. This leaves a significant gap in dementia epidemiology for Asian Americans, especially when disaggregated by ethnic background. This study aims to compare sex/gender differences in lifetime dementia risk, considering death prior to dementia diagnosis as a competing event, among Chinese, Filipino, Japanese, South Asian, and non-Latino White adults aged 60 and older in Northern California.

## Results

### Cohort description

The study included 159,477 participants: 6415 Chinese, 5020 Filipino, 3314 Japanese, 1061 South Asian, and 143,667 non-Latino White (Table 1); the proportion of women ranged from 39% (South Asian) to 63% (Japanese). Mean baseline age was similar for women and men, ranging from 69 years among South Asians to 73 years among Japanese (age distribution in Supplementary Information, Fig. S1). The percentage of participants born in the U.S. varied by Asian American ethnicity and sex/gender: among Chinese and South Asian participants, a higher percentage of women versus men were U.S.-born (34% vs. 28% and 10% vs. 5%, respectively), among Filipino participants, the percentage of U.S.-born participants was the same for women and men (10%), and among Japanese participants, a lower percentage of women versus men were U.S.-born (62% vs. 87%). Women tended to have lower educational attainment than men, with the largest differences among South Asian and Japanese participants. Across all Asian American ethnic groups, women tended to have lower workforce participation and lower average household size-adjusted income and were more likely to be unmarried and living alone compared to men. Markers of health varied by Asian ethnicity and sex/gender.

**Table 1:** Characteristics of the cohort stratified by race and ethnicity and sex/gender (2002–2020)

	Chinese		Filipino		Japanese		South Asian		Non-Latino White	
Characteristic	Women, *N* = 3245 (51%)	Men, N = 3170 (49%)	Women, *N* = 2850 (57%)	Men, N = 2170 (43%)	Women, *N* = 2079 (63%)	Men, *N* = 1235 (37%)	Women, *N* = 414 (39%)	Men, *N* = 647 (61%)	Women, *N* = 82,895 (58%)	Men, *N* = 60,772 (42%)
Survey age, years	70.6 (7.2)	70.6 (7.2)	69.7 (6.8)	69.1 (6.7)	72.8 (7.5)	72.3 (8.0)	68.2 (6.2)	69.0 (6.5)	71.7 (7.8)	71.3 (7.7)
US-born	1064 (34)	865 (28)	285 (10)	209 (10)	1276 (62)	1050 (87)	40 (10)	29 (5)	74,028 (90)	54,145 (91)
Missing	86	88	61	84	37	22	21	39	1013	1560
Education attainment										
Less than high school diploma	579 (19)	472 (16)	324 (12)	168 (8)	168 (9)	39 (3)	80 (21)	45 (8)	4079 (5)	3142 (6)
High school diploma	517 (17)	355 (12)	297 (11)	212 (10)	511 (26)	184 (16)	54 (14)	45 (8)	16,910 (22)	8,409 (15)
Some college	702 (23)	679 (22)	456 (17)	509 (25)	531 (27)	320 (27)	45 (12)	67 (11)	25,376 (33)	16,041 (28)
College graduate	1282 (42)	1536 (50)	1599 (60)	1159 (57)	721 (37)	634 (54)	206 (54)	440 (74)	30,605 (40)	29,123 (51)
Missing	165	128	174	122	148	58	29	50	5,925	4057
Currently working	776 (24)	870 (28)	1000 (36)	835 (39)	413 (20)	263 (22)	128 (32)	284 (45)	19,008 (23)	15,191 (25)
Missing	49	24	45	30	42	21	8	17	824	511
Household-adjusted income, USD	46,691 (30,091)	47,539 (28,607)	38,096 (25,077)	41,730 (24,338)	52,475 (28,395)	56,787 (27,586)	46,378 (30,526)	52,281 (28,917)	53,774 (29,051)	59,025 (28,119)
Missing	532	328	479	212	437	129	82	62	13,584	6347
Married or living as married	2175 (68)	2769 (88)	1694 (60)	1892 (88)	1209 (59)	971 (79)	294 (72)	565 (89)	45,676 (56)	47,938 (80)
Missing	50	41	27	16	20	8	7	13	921	567
Household size: living alone	604 (19)	252 (8)	307 (11)	115 (5)	574 (29)	189 (16)	55 (14)	40 (6)	26,048 (32)	8922 (15)
Missing	114	106	115	73	72	31	19	27	2647	1621
Self-rated health										
Excellent/very good	811 (26)	743 (27)	801 (29)	496 (26)	390 (19)	237 (21)	96 (24)	116 (20)	13,500 (17)	10,724 (19)
Good	1440 (45)	1283 (46)	1251 (45)	859 (45)	909 (45)	512 (44)	192 (47)	290 (50)	32,733 (40)	23,305 (41)
Fair/Poor	921 (29)	775 (28)	703 (26)	554 (29)	734 (36)	406 (35)	117 (29)	178 (30)	34,640 (43)	22,756 (40)
Missing	73	369	95	261	46	80	9	63	2022	3987
Self-reported depression	205 (6)	137 (4)	203 (7)	106 (5)	136 (7)	52 (4)	37 (9)	40 (6)	12,853 (16)	5852 (10)
BMI	23.9 (3.5)	24.7 (3.2)	25.7 (4.2)	26.2 (3.6)	24.1 (3.8)	25.7 (3.6)	26.2 (4.4)	25.7 (3.9)	27.5 (5.7)	27.9 (4.3)
Missing	650	1467	501	786	505	478	63	237	12,300	20,606
Hypertension	2036 (63)	2015 (64)	2259 (79)	1696 (78)	1382 (66)	854 (69)	267 (64)	449 (69)	53,542 (65)	39,477 (65)
Diabetes	530 (16)	656 (21)	815 (29)	782 (36)	356 (17)	313 (25)	101 (24)	220 (34)	9903 (12)	10630 (17)
Stroke	91 (3)	132 (4)	103 (4)	116 (5)	72 (3)	57 (5)	11 (3)	24 (4)	3221 (4)	3212 (5)
Self-reported depression	205 (6)	137 (4)	203 (7)	106 (5)	136 (7)	52 (4)	37 (9)	40 (6)	12,853 (16)	5852 (10)
Follow-up time	10.2 (3.8)	10.7 (5.3)	9.3 (4.2)	9.2 (5.2)	9.7 (4.1)	10.1 (5.3)	9.9 (4.0)	9.8 (4.9)	9.3 (4.2)	9.3 (4.9)
End of follow-up events										
Dementia	425 (13)	344 (11)	341 (12)	225 (10)	370 (18)	190 (15)	51 (12)	60 (9)	12,666 (15)	8413 (14)
Administratively censored	2037 (63)	1607 (51)	1523 (53)	896 (41)	1156 (56)	584 (47)	239 (58)	307 (47)	42,303 (51)	26,005 (43)
Death	386 (12)	680 (21)	342 (12)	424 (20)	307 (15)	281 (23)	42 (10)	112 (17)	15,242 (18)	15,884 (26)
End of membership	397 (12)	539 (17)	644 (23)	625 (29)	246 (12)	180 (15)	82 (20)	168 (26)	12,684 (15)	10,470 (17)

*Mean (SD) for continuous variables, n(%) for categorical variables. Percentages are calculated excluding missing values. *USD* United States dollars, *BMI* body mass index.

**Fig. 1:**
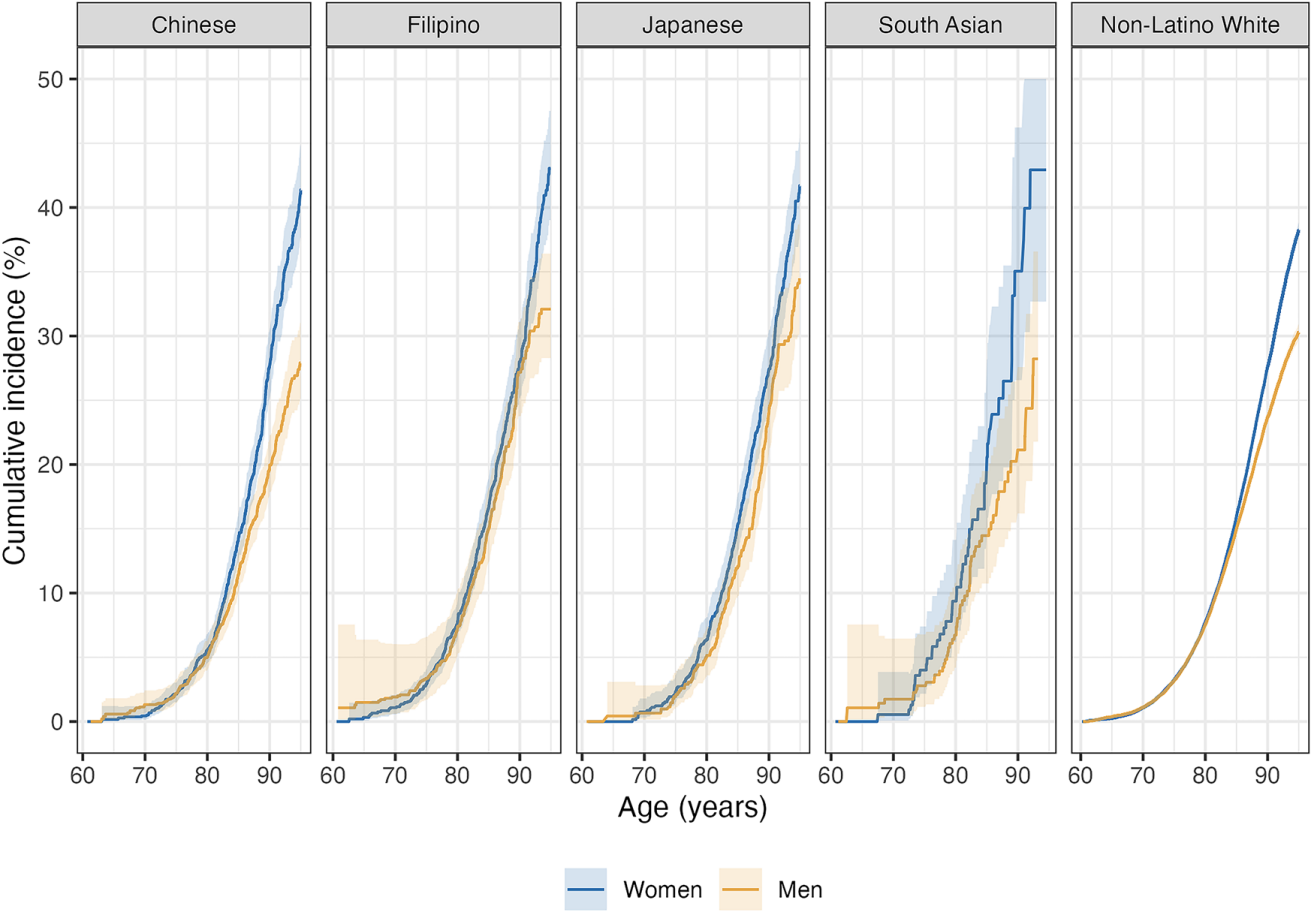
Estimated cause-specific cumulative incidence of dementia from age 60 years to 95 years (95% confidence interval), stratified by race and ethnicity and sex/gender. 95% confidence intervals fully displayed for Chinese, Filipino, Japanese, and Non-Latino White groups. For the South Asian group, the upper bound of the cumulative incidence between ages 91 and 95 was top-coded at 50%; the actual estimated values for the upper bound ranged from 53% to 56%.

Mean follow-up time was 9.4 years (standard deviation = 4.5). Dementia case counts, person-years of follow-up, and age-specific incidence rates per 1000-person years are presented in Supplementary Information, Table S1. Estimated cumulative incidence of dementia (i.e., dementia risk) are presented in Fig. 1 and Supplementary Information, Table S2.

### Dementia risk among women by race and ethnicity

Among women, at age 75, estimated dementia risk was relatively low and similar across Asian ethnic groups, ranging from 2% (95% CI: 1, 3) for Chinese women to 4% (95% CI: 2, 6) for South Asian women. By age 85, estimated dementia risk increased to 14% (95% CI: 12, 16) and 21% (95% CI: 14, 27) for the same groups. Dementia risk increased substantially between ages 85 and 95; estimated lifetime dementia risk (i.e., risk at age 95) ranged from 42% (95% CI: 38, 45) for Chinese women and 42% (95% CI: 38, 46) for Japanese women to 49% (95% CI: 40, 67) for South Asian women. Lifetime dementia risk was lower among non-Latino White women [38% (95% CI: 38, 39)] compared to Asian American women.

### Dementia risk among men by race and ethnicity

Among men, estimated dementia risk at age 75 ranged from 2%–3% in all Asian ethnic groups. By age 85, estimated dementia risk ranged from 11% (95% CI: 10, 13) for Chinese men to 15% (95% CI: 12, 18) for Filipino men. Estimated lifetime dementia risk ranged from 28% (95% CI: 25, 31) for Chinese men and 28% (95% CI: 22, 37) for South Asian men to 34% (95% CI: 30, 39) for Japanese men. Estimated lifetime dementia risk was lower among non-Latino White men (30% (95% CI: 30, 31) than Filipino and Japanese men but higher than among Chinese and South Asian men.

### Sex/gender differences in dementia risk by race and ethnicity

**Fig. 2:**
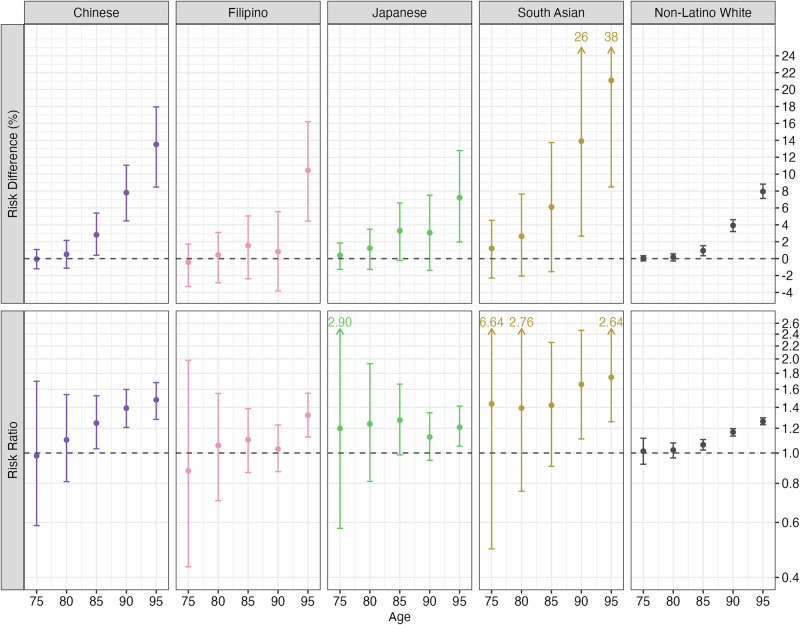
Estimated risk differences and risk ratios (95% confidence interval) relating sex/gender and dementia risk at ages 75, 80, 85, 90, 95 (men as reference group) by race and ethnicity. Risk ratios presented on log scale.

Estimated age-specific risk differences and risk ratios for dementia risk comparing women to men are presented in Fig. 2 and Supplementary Information, Table S2; differences varied by age and across racial and ethnic groups. Among Chinese and non-Latino White participants, differences emerged at age 85 and increased at age 95 on both the absolute and relative scales. Among Filipino participants, differences appeared later, at age 95, on both scales. Among Japanese participants, differences emerged at age 85 on the absolute scale; on the risk ratio scale, estimated risk ratios remained constant at approximately 1.2 between ages 75 and 95, although estimates were imprecise at younger ages. Among South Asian participants, absolute differences emerged at age 80 and increased substantially by age 95, although estimates were imprecise, especially at older ages; on the risk ratio scale, estimated risk ratios remained constant at approximately 1.4 between ages 75 and 85 and increased to approximately 1.7 between ages 90 and 95, although estimates were imprecise, especially at younger ages. Comparing across racial/ethnic groups, lifetime risk differences ranged from 7 (95% CI: 2, 13) percentage points higher for Japanese women compared to men to 21 (95% CI: 8, 38) points higher dementia risk for South Asian women compared to men; lifetime risk ratios ranged from 1.21 (95% CI: 1.05, 1.41) among Japanese participants to 1.75 (95% CI: 1.26, 2.64) among South Asian participants.

### Sex/gender differences in dementia-free mortality by race and ethnicity

**Fig. 3:**
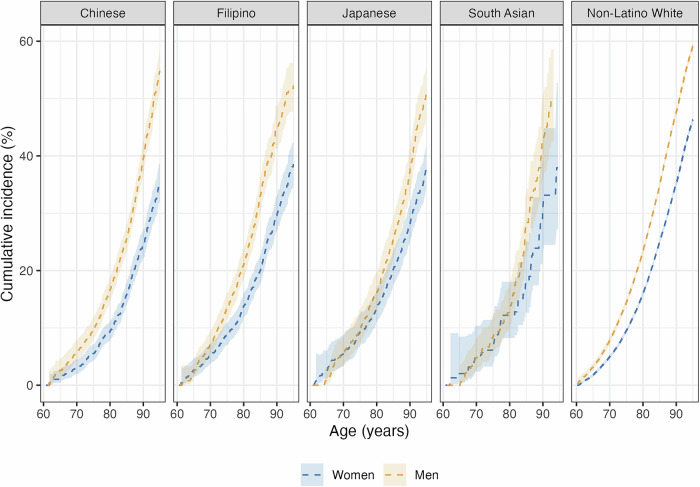
Estimated cause-specific cumulative incidence of dementia-free mortality from age 60 years to 95 years (95% confidence interval), stratified by race and ethnicity and sex/gender. 95% confidence intervals displayed for all groups.

Dementia-free mortality was consistently higher for men than women (Fig. 3; Supplementary Information, Table S3), with sex/gender differences in cumulative dementia-free mortality widening at older ages on both the absolute and relative scales for all racial and ethnic groups (Supplementary Information, Fig. S2). Sex/gender risk differences in dementia-free mortality by age 95 were largest among Chinese participants: dementia-free mortality risk by age 95 among Chinese men exceeded that of women by 20 percentage points (95% CI: 15, 25). Sex/gender risk differences in dementia-free mortality by age 95 were similar among Filipino participants [14% (95% CI: 8, 19)], Japanese participants [14% (95% CI: 9, 20)], non-Latino White participants 13% (95% CI: 12, 14), and South Asian participants [12% (95% CI: −3, 27)].

## Discussion

In this cohort study nested in the Kaiser Permanente Northern California health care system, lifetime dementia risk was higher among women than men, and sex/gender differences emerged at different ages across racial and ethnic groups. Sex/gender differences in lifetime dementia risk were largest among South Asian and Chinese participants. Across all racial and ethnic groups, dementia-free mortality was higher among men than women, with the largest sex/gender differences in dementia-free mortality observed among Chinese older adults.

Our finding that lifetime dementia risk was higher among women versus men is consistent with prior research in White and Black populations in the U.S. that estimated lifetime dementia risk^18,19^. A study from the Framingham Heart Study, which included primarily White adults aged 45 and older with high educational attainment, found that the dementia risk was higher among women than among men from age 85 onwards^18^. Likewise, a recent study of Black and White adults aged 55 and older from the Atherosclerosis Risk in Communities (ARIC) study found that sex differences in dementia risk emerged at approximately age 85 years^19^. These studies used the same methodology as our primary analyses; specifically, both our study and these studies estimate the cause-specific cumulative incidence of dementia, a parameter that accounts for the probability of experiencing the competing event of death^31^. In contrast, parameters that use incidence rates or hazards have an inherent issue of selection bias^32^, which limits their interpretation for meaningful comparisons, especially in settings where competing events such as death are present. This methodological difference could explain why some previous longitudinal studies did not find sex/gender differences in dementia risk^23,25^.

In our study, South Asian older adults, who were predominantly born outside the US, had the largest sex/gender differences in dementia risk on both the absolute and relative scales from age 80 onwards; comparisons across Asian American groups at younger ages are limited given the wide confidence intervals for all groups. South Asian women had the highest lifetime dementia risk compared to Chinese, Filipino, Japanese, and non-Latino White women (Supplementary Information, Table S2). Previous work on Indian older adults in Longitudinal Aging Study of India-Diagnostic Assessment of Dementia (LASI-DAD) showed that women performed significantly worse on cognitive tests compared to men, which is likely attributable to large gender inequities in human capital investment and limited access to formal education for girls in India^20,27^. Similarly, in our study, we observed large sex/gender differences in educational attainment in South Asian participants. This suggests that social and structural factors, which differ across Asian American ethnic groups, contribute to sex/gender differences in lifetime dementia risk.

Chinese older adults, two-thirds of whom were born outside the U.S., had the second largest sex/gender differences in lifetime dementia risk in our study. Previous work on older adults in the Chinese Longitudinal Healthy Longevity Survey found that Chinese women tended to experience faster cognitive decline than men^21^. In our study, Chinese older adults had the largest sex/gender differences in dementia-free mortality by age 95 (Supplementary Information, Table S3 and Fig. S2). These results suggest that differential mortality potentially contributes to sex/gender differences in lifetime dementia risk^14^. A study using data from the U.S. mortality records from the National Center for Health Statistics (NCHS) from 2003–2011 found sex/gender differences on the leading causes of death within and between Asian American ethnic groups^33^.

One major limitation is that we could not distinguish sex from gender based on available EHR information. In addition, we were unable to disaggregate the national identities within the South Asian population, as the survey grouped all South Asians into a single category, combining heterogeneous populations with diverse ethnic and cultural backgrounds. Based on the distribution of South Asians in Northern California, this group is most likely represented by older adults of Indian origin, with smaller representation of older adults from Pakistan, Bangladesh, Nepal, and Sri Lanka^34^. Considering that South Asian women had the highest lifetime dementia risk in our study, it is important for future work on dementia to disaggregate South Asian adults. To our knowledge, this study provides the largest source of evidence on sex/gender differences in lifetime dementia risk among Asian Americans in the U.S. However our sample over-represents U.S.-born Asian American individuals, is healthier, and likely under-represents non-English speakers compared to the broader KPNC membership and the California general population of Asian American older adults^7,35^. These estimates may not be generalizable to Asian Americans living in other states, which have varying patterns of ethnic group composition and distribution of Asian American ethnic enclaves, as well as varying health care access^36^. Lastly, we obtained dementia diagnosis from the EHR, which could underestimate dementia incidence overall. There is mixed evidence as to whether timeliness of dementia diagnosis differs among Asian Americans compared with other racial and ethnic groups^37,38^. Timeliness of diagnosis could vary across Asian ethnic groups^37^, and since Asian American women face more barriers in healthcare^11,12^, it is possible that there are greater delays in dementia diagnosis among women than men, which could lead to underestimation of sex/gender differences in dementia risk.

To conclude, lifetime dementia risk was higher among women than men, but sex/gender differences emerged at different ages across racial and ethnic groups. Higher mortality among men contributed to higher lifetime dementia risk among women, and our findings are consistent with higher burden of social and structural risk factors contributing to higher dementia risk among women than men. This is the first comprehensive study on lifetime dementia risk across Asian American ethnicities and the first to estimate sex/gender differences in dementia risk among Asian Americans—a group that has been understudied in dementia research. Future research should focus on evaluating the extent to which intersectional structural and social factors, as well as genetic factors, place Asian American women at higher risk of dementia compared to Asian American men. Furthermore, replication of our work in other settings is much needed, which requires the systematic collection of granular information on race, ethnicity, sex, and gender to provide meaningful and informative estimates.

## Methods

### Data source

We included data from a cohort of Asian American and non-Latino White members of Kaiser Permanente Northern California (KPNC) who completed one of two harmonized health surveys: the California Men’s Health Study (CMHS, administered 2002–2003)^39^ or the Kaiser Permanente Research Program on Genes, Environment, and Health Survey (RPGEH, administered 2007–2009)^40,41^. KPNC implemented electronic health records (EHR) in January 1996. The analytic sample included participants who were ages 60–90 years with at least 2 years of continuous KPNC membership and no prior EHR diagnosis of dementia prior to their survey date (study baseline). We included participants from ethnic groups with at least 5 dementia cases in each age interval, stratified by sex/gender (Chinese, Filipino, Japanese, South Asian, or non-Latino White).

Informed consent for survey participation was obtained by KPNC at the time of the survey completion; analysis of the de-identified data was approved by the University of California, Los Angeles Institutional Review Board (#19-000794).

### **Variable definitions**

Race and ethnicity were self-reported defined based on the survey question that asked participants to select all groups that best described their race or ethnicity. Asian American participants were classified by the Asian ethnicity they reported: Chinese, Japanese, South Asian (“South Asian: Indian, Pakistani, etc.” in the RPGEH survey and “Asian Indian” in the CMHS survey), Korean, Vietnamese, or Other Southeast Asian. Participants who reported only “White or European-American” were considered non-Latino White. Our primary focus was on Asian American ethnic groups, but we included non-Latino White participants because most of the literature on this topic includes predominantly non-Latino White individuals. Our motivation was that if our results for non-Latino White participants aligned with prior literature, that would increase our confidence in results for the Asian American ethnic groups.

Sex was derived from EHR; data on patient-reported gender identity were unavailable. We acknowledge that sex is a biological construct while gender refers to social, cultural, and psychological traits linked to individuals through social context^15^. In this work, we use the term “sex/gender” to acknowledge that the variable labeled as “sex” likely reflects a combination of sex and gender^42^.

Incident dementia diagnoses were identified from the EHR using International Classification of Disease (ICD) 9th and 10th Revision codes for Alzheimer’s disease, vascular dementia, and non-specific dementia (Supplementary Information, Table S4). A minimum of one inpatient or outpatient diagnosis or two telephone encounters was required for a dementia diagnosis. Deaths were identified from the KPNC mortality database, which aggregates KPNC clinical and administrative sources, the National Death Index, California State death records, and Social Security Administration records. We followed participants to dementia diagnosis, death, lapse in health plan coverage ≥90 days (loss to follow-up), or end of the study period (March 2020). All events occurring at age 90 and older were top-coded to de-identify the data; we used imputed event times at age 90 and older as previously reported^35^.

To describe the sample, we included the following baseline characteristics derived from surveys: U.S. nativity (born in the U.S. yes/no), educational attainment (less than high school diploma, high school diploma, some college, college graduate), current employment status (currently working yes/no; “no” included retired, disabled, full-time student, homemaker, unemployed, or other), household-size adjusted income (U.S. dollars), marital status (married or living as married: yes/no), living alone (yes/no), self-rated health (excellent/very good, good, fair/poor), and self-reported depression (yes/no). Baseline body mass index (BMI), history of hypertension, history of diabetes, and history of stroke were derived from EHR. Annual median values of height and weight at baseline were used to calculate BMI. Diabetes status was identified from the Kaiser Diabetes Registry^43,44^. Hypertension and stroke were identified from the EHR based on ICD codes (Supplementary Information, Table S5).

### Statistical analysis

All analyses were stratified by race and ethnicity and sex/gender. First, we estimated age-specific dementia incidence rates (≦74, 75–79, 80–84, 85–89, 90–94, 95+ years). Next, we used an Aalen-Johansen estimator with age as the timescale to calculate the cause-specific cumulative incidence of dementia (i.e., dementia risk) starting at age 60 years, accounting for left truncation for baseline ages over 60 years and treating death as a competing event, which sets the risk of dementia to zero upon a participant’s death. We defined lifetime dementia risk as the cause-specific cumulative incidence of dementia by age 95. We calculated risk differences and risk ratios at ages 75, 80, 85, 90, and 95 (men as the reference group); 95% confidence intervals were estimated with 1000 bootstraps. Since sex/gender differences in mortality contribute to sex/gender differences in dementia risk^45^, we additionally calculated cumulative incidence of dementia-free mortality for women and men and corresponding risk differences and risk ratios. We did not adjust for covariates since we conceptualize them all as potential mediators in the association between sex/gender and dementia risk.

Analyses were conducted using R statistical software version 4.4.1; cause-specific cumulative incidence of dementia and death were calculated using the ‘survival’ package.

## Supplementary information

Supplementary Information

## Data Availability

The data underlying this article were provided by the Kaiser Permanente Research Bank. Information on the application process for researchers interested in using Kaiser Permanente Research Bank data can be found at https://researchbank.kaiserpermanente.org/our-research/for-researchers/.

## Code availability

The underlying code for this study is available in Github and can be accessed via https://github.com/Mayeda-Research-Group/aa_sex_gender_dem.
