# Supplementary material for: Sex/gender differences in lifetime dementia risk among Asian American and White older adults
Source: NPJ Dement. 2025 Oct 13;1(1):32. doi: 10.1038/s44400-025-00038-8 (PMC12518132; doi:10.1038/s44400-025-00038-8)
Supplement: Supplementary file 1 — Supplementary Information [file 44400_2025_38_MOESM1_ESM.docx]

**Sex/Gender Differences in Lifetime Dementia Risk Among Asian American and White Older Adults**

L. Paloma Rojas-Saunero, Yingyan Wu, Yixuan Zhou, Eleanor Hayes-Larson, Gilbert C. Gee, Ron Brookmeyer, Holly Elser, Alexander Ivan B. Posis, Alka M. Kanaya, Rachel A. Whitmer, Paola Gilsanz, Elizabeth Rose Mayeda

**Supplementary Information**

[Supplementary results 2](#_Toc209456068)

[Figure S1. Density plot of baseline age by race and ethnicity and sex/gender 2](#_Toc209456069)

[Table S1. Age-specific dementia incidence rates per 1,000 person-years by race and ethnicity and sex/gender. 3](#_Toc209456070)

[Table S2. Estimated cumulative incidence of dementia by age, race and ethnicity, and sex/gender, with risk differences and risk ratios comparing women vs. men. 4](#_Toc209456071)

[Table S3. Estimated cumulative incidence of dementia-free mortality by age, race and ethnicity, and sex/gender, with risk differences and risk ratios comparing women vs. men. 5](#_Toc209456072)

[Figure S2. Estimated risk differences and risk ratios (95% confidence interval) relating sex/gender and dementia-free mortality at ages 75, 80, 85, 90, 95 (men as the reference group) by race and ethnicity. Risk ratios presented on the log scale. 6](#_Toc209456073)

[Detailed information on variables included in the study 7](#_Toc209456074)

[Table S4. International Classification of Disease, Ninth Edition, Clinical Modification (ICD-9-CM) and International Classification of Disease, Tenth Edition, Clinical Modification (ICD-10-CM) codes used to identify dementia (Alzheimer’s disease, vascular dementia, and non-specific dementia) 7](#_Toc209456075)

[Table S5. International Classification of Disease, Ninth Edition, Clinical Modification (ICD-9-CM) and International Classification of Disease, Tenth Edition, Clinical Modification (ICD-10-CM) codes used to identify stroke and hypertension 8](#_Toc209456076)

# Supplementary results

## Figure S1. Density plot of baseline age by race and ethnicity and sex/gender


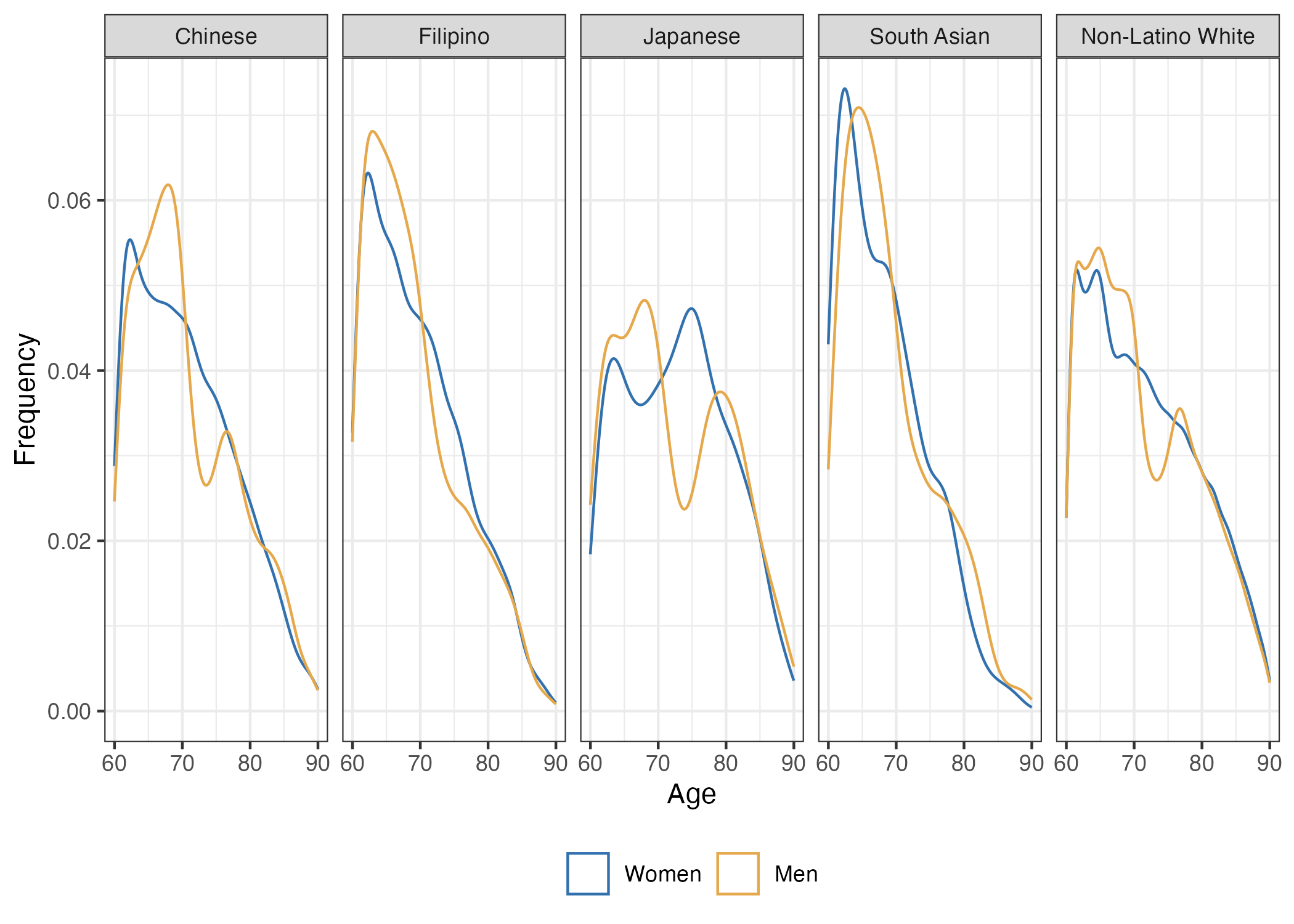


## Table S1. Age-specific dementia incidence rates per 1,000 person-years by race and ethnicity and sex/gender.

| **Race/ethnicity** | **Age range** | **Cases** | | **Person-Years** | | **Incidence Rates (95% CI)** | |
| --- | --- | --- | --- | --- | --- | --- | --- |
|  |  | **Women** | **Men** | **Women** | **Men** | **Women** | **Men** |
| Chinese |  |  |  |  |  |  |  |
|  | <= 74 years old | 34 | 29 | 16288.8 | 15746.1 | 2.1 (1.4, 2.8) | 1.8 (1.2, 2.5) |
|  | 75-79 years old | 57 | 56 | 7423.1 | 8177.9 | 7.7 (5.7, 9.7) | 6.8 (5.1, 8.6) |
|  | 80-84 years old | 118 | 109 | 5431.6 | 6154.7 | 21.7 (17.8, 25.6) | 17.7 (14.4, 21.0) |
|  | 85-89 years old | 125 | 94 | 2923.8 | 2952.6 | 42.8 (35.3, 50.2) | 31.8 (25.4, 38.3) |
|  | 90-94 years old | 73 | 46 | 971.2 | 858.0 | 75.2 (57.9, 92.4) | 53.6 (38.1, 69.1) |
|  | 95+ years | 18 | 10 | 122.8 | 101.3 | 146.6 (78.9, 214.3) | 98.7 (37.5, 159.9) |
| Filipino |  |  |  |  |  |  |  |
|  | <= 74 years old | 38 | 25 | 14355.8 | 11129.6 | 2.6 (1.8, 3.5) | 2.2 (1.4, 3.1) |
|  | 75-79 years old | 68 | 44 | 5897.7 | 4459.2 | 11.5 (8.8, 14.3) | 9.9 (7.0, 12.8) |
|  | 80-84 years old | 95 | 68 | 3876.6 | 2842.8 | 24.5 (19.6, 29.4) | 23.9 (18.2, 29.6) |
|  | 85-89 years old | 81 | 67 | 1873.8 | 1192.2 | 43.2 (33.8, 52.6) | 56.2 (42.7, 69.7) |
|  | 90-94 years old | 54 | 16 | 561.5 | 317.8 | 96.2 (70.5, 121.8) | 50.3 (25.7, 75.0) |
|  | 95+ years | 5 | 5 | 74.0 | 53.2 | 67.6 (8.3, 126.8) | 94.0 (11.6, 176.5) |
| Japanese |  |  |  |  |  |  |  |
|  | <= 74 years old | 20 | 10 | 7955.8 | 5356.7 | 2.5 (1.4, 3.6) | 1.9 (0.7, 3.0) |
|  | 75-79 years old | 44 | 21 | 4578.2 | 2713.9 | 9.6 (6.8, 12.5) | 7.7 (4.4, 11.0) |
|  | 80-84 years old | 98 | 47 | 4026.1 | 2403.7 | 24.3 (19.5, 29.2) | 19.6 (14.0, 25.1) |
|  | 85-89 years old | 110 | 67 | 2520.9 | 1500.0 | 43.6 (35.5, 51.8) | 44.7 (34.0, 55.4) |
|  | 90-94 years old | 75 | 38 | 890.1 | 481.4 | 84.3 (65.2, 103.3) | 78.9 (53.8, 104.0) |
|  | 95+ years | 23 | 7 | 124.4 | 53.8 | 185.0 (109.4, 260.5) | 130.1 (33.7, 226.4) |
| South Asian |  |  |  |  |  |  |  |
|  | <= 74 years old | 10 | 7 | 2479.9 | 3494.8 | 4.0 (1.5, 6.5) | 2.0 (0.5, 3.5) |
|  | 75-79 years old | 11 | 13 | 855.8 | 1441.6 | 12.9 (5.3, 20.5) | 9.0 (4.1, 13.9) |
|  | 80-84 years old | 14 | 21 | 508.5 | 912.0 | 27.5 (13.1, 42.0) | 23.0 (13.2, 32.9) |
|  | 85-89 years old | 11 | 10 | 190.1 | 366.5 | 57.9 (23.7, 92.1) | 27.3 (10.4, 44.2) |
|  | 90-94 years old | <5 | 6 | 41.1 | 110.7 | < 5 events | 54.2 (10.8, 97.5) |
|  | 95+ years | <5 | <5 | 7.5 | 13.2 | < 5 events | < 5 events |
| Non-Latino White |  |  |  |  |  |  |  |
|  | <= 74 years old | 1156 | 868 | 364257.3 | 273156.3 | 3.2 (3.0, 3.4) | 3.2 (3.0, 3.4) |
|  | 75-79 years old | 1803 | 1450 | 166107.1 | 126785.2 | 10.9 (10.4, 11.4) | 11.4 (10.8, 12.0) |
|  | 80-84 years old | 3079 | 2339 | 128285.4 | 94606.6 | 24.0 (23.2, 24.8) | 24.7 (23.7, 25.7) |
|  | 85-89 years old | 3828 | 2327 | 80170.6 | 53156.7 | 47.7 (46.2, 49.3) | 43.8 (42.0, 45.6) |
|  | 90-94 years old | 2301 | 1198 | 28904.1 | 17737.5 | 79.6 (76.4, 82.9) | 67.5 (63.7, 71.4) |
|  | 95+ years | 499 | 231 | 4021.6 | 2284.5 | 124.1 (113.2, 135.0) | 101.1 (88.1, 114.2) |

## Table S2. Estimated cumulative incidence of dementia by age, race and ethnicity, and sex/gender, with risk differences and risk ratios comparing women vs. men.

| **Race and Ethnicity** | **Age** | **Cumulative**  **incidence (%) for women (95% CI)** | **Cumulative**  **incidence (%) for men (95% CI)** | **Risk difference (%) (95% CI)** | **Risk ratio**  **(95% CI)** |
| --- | --- | --- | --- | --- | --- |
| Chinese | 75 | 2 (1, 3) | 2 (1, 3) | 0 (-1, 1) | 0.98 (0.59, 1.70) |
|  | 80 | 6 (4, 7) | 5 (4, 6) | 1 (-1, 2) | 1.10 (0.81, 1.54) |
|  | 85 | 14 (12, 16) | 11 (10, 13) | 3 (0, 5) | 1.25 (1.03, 1.52) |
|  | 90 | 28 (25, 30) | 20 (18, 22) | 8 (4, 11) | 1.39 (1.21, 1.60) |
|  | 95 | 42 (38, 45) | 28 (25, 31) | 14 (8, 18) | 1.48 (1.28, 1.68) |
| Filipino | 75 | 3 (2, 4) | 3 (2, 6) | 0 (-3, 2) | 0.88 (0.43, 1.98) |
|  | 80 | 8 (6, 9) | 7 (5, 10) | 0 (-3, 3) | 1.06 (0.70, 1.55) |
|  | 85 | 17 (15, 19) | 15 (12, 18) | 2 (-2, 5) | 1.10 (0.87, 1.39) |
|  | 90 | 28 (25, 31) | 27 (24, 31) | 1 (-4, 6) | 1.03 (0.87, 1.23) |
|  | 95 | 43 (39, 47) | 33 (29, 37) | 10 (4, 16) | 1.32 (1.12, 1.55) |
| Japanese | 75 | 2 (1, 3) | 2 (1, 3) | 0 (-1, 2) | 1.20 (0.57, 2.90) |
|  | 80 | 6 (5, 8) | 5 (3, 7) | 1 (-1, 3) | 1.24 (0.81, 1.93) |
|  | 85 | 15 (13, 17) | 12 (10, 15) | 3 (0, 7) | 1.27 (0.99, 1.66) |
|  | 90 | 27 (25, 30) | 24 (21, 28) | 3 (-1, 8) | 1.13 (0.95, 1.34) |
|  | 95 | 42 (38, 46) | 34 (30, 39) | 7 (2, 13) | 1.21 (1.05, 1.41) |
| South Asian | 75 | 4 (2, 6) | 3 (1, 5) | 1 (-2, 5) | 1.44 (0.49, 6.64) |
|  | 80 | 9 (6, 13) | 7 (4, 10) | 3 (-2, 8) | 1.39 (0.75, 2.76) |
|  | 85 | 21 (14, 27) | 14 (10, 19) | 6 (-2, 14) | 1.42 (0.91, 2.26) |
|  | 90 | 35 (26, 46) | 21 (15, 27) | 14 (3, 26) | 1.66 (1.11, 2.47) |
|  | 95 | 49 (40, 67) | 28 (22, 37) | 21 (8, 38) | 1.75 (1.26, 2.64) |
| Non-Latino White | 75 | 3 (3, 3) | 3 (3, 3) | 0 (0, 0) | 1.01 (0.92, 1.12) |
|  | 80 | 8 (7, 8) | 8 (7, 8) | 0 (0, 1) | 1.02 (0.96, 1.08) |
|  | 85 | 16 (16, 16) | 15 (15, 15) | 1 (0, 2) | 1.06 (1.02, 1.10) |
|  | 90 | 28 (27, 28) | 24 (23, 24) | 4 (3, 5) | 1.17 (1.13, 1.20) |
|  | 95 | 38 (38, 39) | 30 (30, 31) | 8 (7, 9) | 1.26 (1.23, 1.30) |

## Table S3. Estimated cumulative incidence of dementia-free mortality by age, race and ethnicity, and sex/gender, with risk differences and risk ratios comparing women vs. men.

| **Race and**  **Ethnicity** | **Age** | **Cumulative**  **incidence (%) for women (95% CI)** | **Cumulative**  **incidence (%) for men (95% CI)** | **Risk difference (%) (95% CI)** | **Risk ratio**  **(95% CI)** |
| --- | --- | --- | --- | --- | --- |
| Chinese | 75 | 6 (4, 7) | 10 (8, 12) | -4 (-6, -2) | 0.57 (0.42, 0.80) |
|  | 80 | 9 (8, 11) | 17 (14, 19) | -7 (-10, -4) | 0.57 (0.46, 0.70) |
|  | 85 | 16 (14, 18) | 26 (23, 28) | -10 (-13, -7) | 0.61 (0.53, 0.72) |
|  | 90 | 25 (22, 27) | 40 (37, 42) | -15 (-19, -11) | 0.62 (0.55, 0.70) |
|  | 95 | 35 (32, 39) | 55 (51, 58) | -20 (-25, -15) | 0.64 (0.57, 0.71) |
| Filipino | 75 | 9 (7, 11) | 14 (11, 16) | -5 (-8, -2) | 0.64 (0.50, 0.83) |
|  | 80 | 14 (12, 16) | 21 (18, 24) | -7 (-11, -4) | 0.66 (0.54, 0.80) |
|  | 85 | 20 (18, 23) | 33 (30, 36) | -13 (-17, -9) | 0.61 (0.52, 0.71) |
|  | 90 | 30 (27, 33) | 45 (41, 49) | -15 (-20, -10) | 0.66 (0.58, 0.77) |
|  | 95 | 39 (34, 43) | 52 (48, 57) | -14 (-19, -8) | 0.74 (0.65, 0.84) |
| Japanese | 75 | 9 (7, 12) | 10 (7, 13) | -1 (-4, 3) | 0.95 (0.64, 1.45) |
|  | 80 | 14 (11, 17) | 16 (13, 20) | -2 (-6, 2) | 0.85 (0.65, 1.13) |
|  | 85 | 20 (17, 23) | 25 (22, 29) | -5 (-10, -1) | 0.79 (0.65, 0.97) |
|  | 90 | 28 (25, 31) | 37 (33, 42) | -9 (-15, -5) | 0.75 (0.64, 0.87) |
|  | 95 | 38 (34, 41) | 51 (47, 56) | -14 (-20, -9) | 0.73 (0.64, 0.82) |
| South Asian | 75 | 7 (4, 11) | 9 (5, 12) | -2 (-7, 3) | 0.82 (0.37, 1.53) |
|  | 80 | 12 (7, 17) | 13 (10, 17) | -1 (-7, 5) | 0.92 (0.54, 1.40) |
|  | 85 | 18 (12, 24) | 27 (23, 33) | -10 (-18, -2) | 0.65 (0.42, 0.91) |
|  | 90 | 33 (23, 44) | 43 (36, 51) | -10 (-22, 3) | 0.77 (0.53, 1.07) |
|  | 95 | 38 (26, 50) | 50 (41, 59) | -12 (-27, 3) | 0.76 (0.49, 1.06) |
| Non-Latino White | 75 | 10 (9, 10) | 14 (14, 15) | -5 (-6, -4) | 0.66 (0.62, 0.70) |
|  | 80 | 16 (16, 16) | 23 (23, 24) | -7 (-8, -7) | 0.68 (0.66, 0.71) |
|  | 85 | 25 (25, 25) | 35 (34, 36) | -10 (-11, -9) | 0.72 (0.70, 0.74) |
|  | 90 | 35 (35, 36) | 48 (47, 48) | -12 (-13, -12) | 0.74 (0.72, 0.76) |
|  | 95 | 46 (46, 47) | 59 (59, 60) | -13 (-14, -12) | 0.78 (0.77, 0.80) |

## Figure S2. Estimated risk differences and risk ratios (95% confidence interval) relating sex/gender and dementia-free mortality at ages 75, 80, 85, 90, 95 (men as the reference group) by race and ethnicity. Risk ratios presented on the log scale.


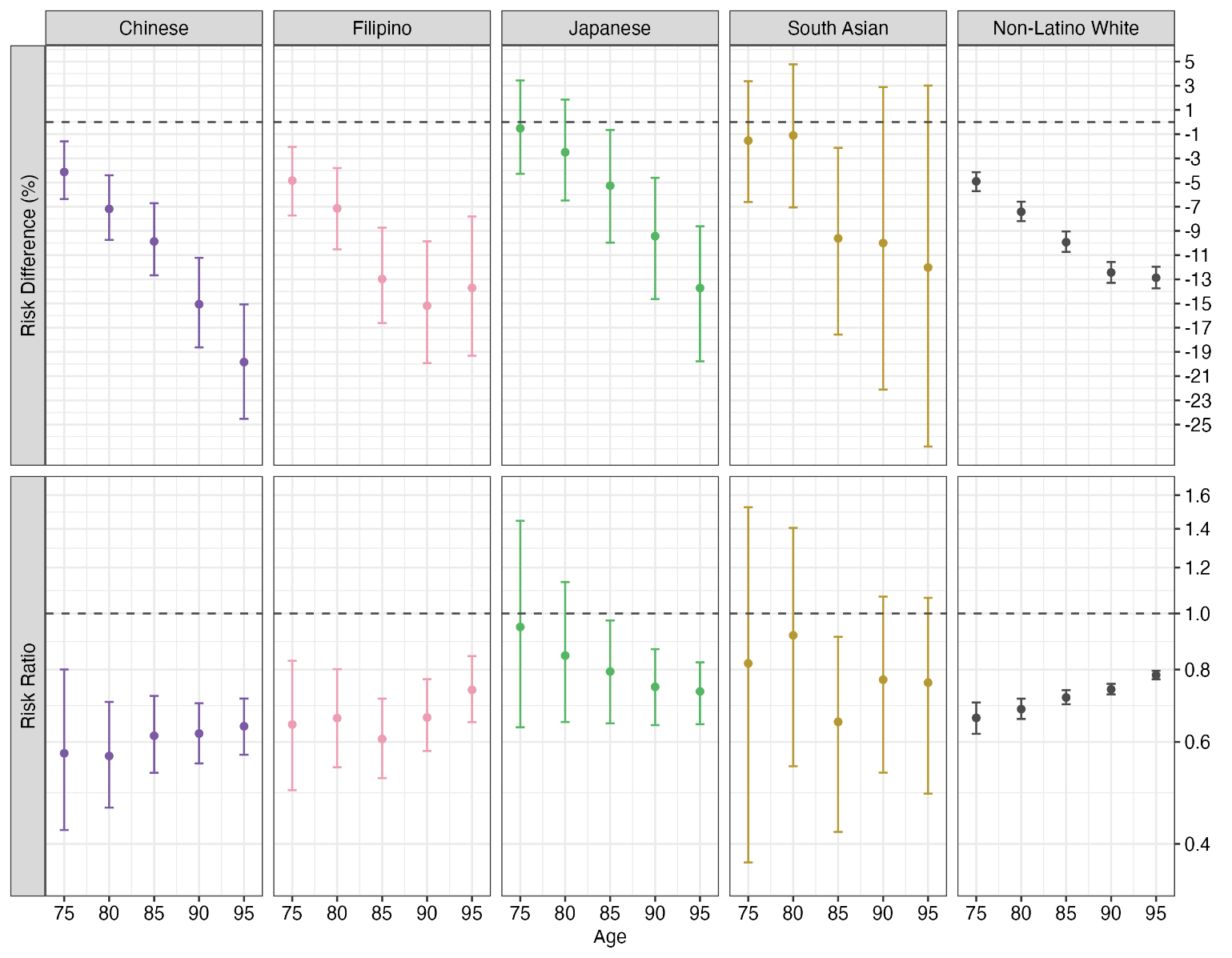


# Detailed information on variables included in the study

## Table S4. International Classification of Disease, Ninth Edition, Clinical Modification (ICD-9-CM) and International Classification of Disease, Tenth Edition, Clinical Modification (ICD-10-CM) codes used to identify dementia (Alzheimer’s disease, vascular dementia, and non-specific dementia)

|  | ICD Code | Description |
| --- | --- | --- |
| ICD-9-CM | 331.0 | Alzheimer's disease |
|  | 290.40 | Vascular dementia, uncomplicated |
|  | 290.41 | Vascular dementia, with delirium |
|  | 290.42 | Vascular dementia, with delusions |
|  | 290.43 | Vascular dementia, with depressed mood |
|  | 290.0 | Senile dementia, uncomplicated |
|  | 290.10 | Presenile dementia, uncomplicated |
|  | 290.11 | Presenile dementia with delirium |
|  | 290.12 | Presenile dementia with delusional features |
|  | 290.13 | Presenile dementia with depressive features |
|  | 290.3 | Senile dementia with delirium |
|  | 294.20 | Dementia, unspecified, without behavioral disturbance |
|  | 294.21 | Dementia, unspecified, with behavioral disturbance |
|  | 294.8 | Other persistent mental disorders due to conditions classified elsewhere |
| ICD-10-CM | G30.0 | Alzheimer's Disease with early onset |
|  | G30.1 | Alzheimer's disease with late onset |
|  | G30.8 | Other Alzheimer's disease |
|  | G30.9 | Alzheimer's disease, unspecified |
|  | F01.50 | Vascular dementia without behavioral disturbance |
|  | F01.51 | Vascular dementia with behavioral disturbance |
|  | F03.90 | Unspecified Dementia without behavioral disturbance |
|  | F03.91 | Unspecified Dementia with behavioral disturbance |

## Table S5. International Classification of Disease, Ninth Edition, Clinical Modification (ICD-9-CM) and International Classification of Disease, Tenth Edition, Clinical Modification (ICD-10-CM) codes used to identify stroke and hypertension

| **Variable Name** | | **ICD-9 diagnosis codes** | **ICD-10 diagnosis codes** |
| --- | --- | --- | --- |
| Combined stroke | Ischemic stroke | 433.01 Occlusion and stenosis of basilar artery with cerebral infarction 433.11 Occlusion and stenosis of carotid artery with cerebral infarction  433.21 Occlusion and stenosis of vertebral artery with cerebral infarction 433.31 Occlusion and stenosis of multiple and bilateral precerebral arteries with cerebral infarction) 433.81 Occlusion and stenosis of other specified precerebral artery with cerebral infarction  433.91 Occlusion and stenosis of unspecified precerebral artery with cerebral infarction 434.xx Occlusion of cerebral arteries | I63.xxx Cerebral Infarction I66.xxx Occlusion and stenosis of cerebral arteries, not resulting in cerebral infarction |
|  | Acute ill-defined cerebrovascular disease (included in definition of ischemic stroke) | 436 Acute but ill-defined cerebrovascular disease | I67.89 Other cerebrovascular disease |
|  | Hemorrhagic stroke | 430 Subarachnoid hemorrhage 431 Intracerebral hemorrhage 432 Other and unspecified intracranial hemorrhage | I60.xx Nontraumatic subarachnoid hemorrhage I61.xx Nontraumatic intracerebral hemorrhage I62.xx Other and unspecified nontraumatic intracranial hemorrhage |
| Hypertension | | 362.11 (Hypertensive retinopathy) 401.x-404.xx (Hypertensive Disease)  437.2 (Hypertensive encephalopathy) | I10 (Essential (primary) hypertension) I11 Hypertensive heart disease I12 Hypertensive chronic kidney disease I13 Hypertensive heart and chronic kidney disease I16 Hypertensive crisis H35.03x (Hypertensive retinopathy) I67.4 (Hypertensive encephalopathy) |
